# Neuroinflammation in Alzheimer’s disease: microglial signature and their relevance to disease

**DOI:** 10.1186/s41232-023-00277-3

**Published:** 2023-05-10

**Authors:** Akira Sobue, Okiru Komine, Koji Yamanaka

**Affiliations:** 1 Department of Neuroscience and Pathobiology, Research Institute of Environmental Medicine, Nagoya University, Aichi, 464-8601 Japan; 2 Department of Neuroscience and Pathobiology, Nagoya University Graduate School of Medicine, Aichi, 466-8550 Japan; 3 Medical Interactive Research and Academia Industry Collaboration Center, Research Institute of Environmental Medicine, Nagoya University, Aichi, 464-8601 Japan; 4 Institute for Glyco-core Research (iGCORE), Nagoya University, Aichi, Japan; 5 Center for One Medicine Innovative Translational Research (COMIT), Nagoya University, Aichi, Japan

**Keywords:** Alzheimer's disease, Disease-associated microglia, Homeostatic microglia, Neuroinflammation, Precuneus

## Abstract

Alzheimer’s disease (AD) is the most common form of dementia, pathologically characterized by senile plaques and neurofibrillary tangles (NFTs), resulting in neurodegeneration. Neuroinflammation, defined as the activation of glial cells such as microglia and astrocytes, is observed surrounding senile plaques and affected neurons in AD. Recently conducted genome-wide association studies (GWAS) indicate that a large section of identified AD risk genes are involved in immune responses and are enriched in microglia. Microglia are innate immune cells in the central nervous system (CNS), which are involved in immune surveillance and maintenance of homeostasis in the CNS. Recently, a novel subpopulation of activated microglia named as disease-associated microglia (DAM), also known as activated response microglia (ARM) or microglial neurodegenerative phenotype (MGnD), was identified in AD model mice. These microglia closely associate with *β*-amyloid (Aβ) plaques and exhibit characteristic gene expression profiles accompanied with reduced expressions of homeostatic microglial genes. However, it remains unclear whether decreased homeostatic microglia functions or increased DAM/ARM/MGnD functions correlate with the degree of neuronal loss in AD. To translate the results of rodent studies to human AD, precuneus, the brain region vulnerable to *β*-amyloid accumulation in preclinical AD, is of high interest, as it can provide novel insights into the mechanisms of microglia response to Aβ in early AD. In this study, we performed comparative analyses of gene expression profiles of microglia among three representative neurodegenerative mouse models and the human precunei with early AD pathology. We proceeded to evaluate the identified genes as potential therapeutic targets for AD. We believe that our findings will provide important resources to better understand the role of glial dysfunction in AD.

## Background

### Neuroinflammation and microglia in Alzheimer’s disease (AD)

AD is the most common cause of dementia and is associated with a progressive neurodegeneration. Globally, the number of patients with dementia is predicted to reach 139 million in 2050 [1]. AD is pathologically characterized by senile plaques and intracellular neurofibrillary tangles (NFTs), consisting of *β*-amyloid (Aβ) aggregates and hyperphosphorylated microtubule-associated protein Tau, respectively, resulting in neuronal dystrophy and loss, respectively [2]. Neuroinflammation, defined as activation of glial cells, such as microglia and astrocytes, and subsequent production of inflammatory factors such as cytokines and chemokines surrounding senile plaques and affected neurons in the brains, is observed in AD patients [3]. Genome-wide association studies (GWAS) of AD risk genetic variants revealed that a large proportion of identified genes were closely related to immune responses, and that their expressions were enriched in microglia and macrophages [4–7].

Microglia, one of the resident innate immune cells in the central nervous system (CNS), originate from erythromyeloid progenitor cells in the embryonic yolk sac [8]. Microglia play an important role in immune surveillance, by phagocytotic clearance of pathogens, dead cells, cellular debris, and protein aggregates like those of Aβ, and help maintain homeostasis in the CNS [9]. Microglia also contribute to brain development and its maintenance by participating in synaptic pruning and myelination [10]. Meanwhile, once microglia respond to their stimuli, their gene expression profiles undergo distinct alterations, with an immediate production of various inflammatory cytokines and mediators and a change in their morphology to an amoeboid shape [11]. It is suggested that long-lasting neuroinflammation causes a decline in homeostatic functions of microglia, resulting in neuronal loss and neurodegenerative diseases. However, it is unknown whether the loss of homeostatic functions of microglia can be correlated with the degree of neurodegeneration and neuronal loss.

Recent advanced single-cell technologies have revealed that microglia intrinsically are a heterogeneous population. One group identified a novel subpopulation of activated microglia named as disease-associated microglia (DAM) or activated response microglia (ARM) in AD model mice [12, 13]. Another group also identified a similar microglia subpopulation, namely microglial neurodegenerative phenotype (MGnD) by RNA sequence (RNA-seq) analysis of isolated microglia from the AD model mice [14]. These microglial populations were observed to be associated with Aβ plaques, displayed characteristic gene expression profiles, and were accompanied with decreased expressions of homeostatic microglia marker genes. Moreover, these microglia were also identified in a mouse model of amyotrophic lateral sclerosis (ALS), a neurodegenerative disease characterized by a selective loss of motor neurons. They expressed an ALS-linked mutant form of superoxide dismutase 1 (*SOD1*) [12, 14]. Intriguingly, whereas *TREM2* (triggering receptor expressed on myeloid cells 2) and *APOE* (apolipoprotein E) are major AD risk genes [15–17], the expressions of TREM2 and APOE in microglia were found to be necessary for the induction of DAM/ARM/MGnD in both AD and ALS model mice [12–14]. However, it is still not clear whether the contribution is either positive or negative of DAM/ARM/MGnD in AD and ALS.

### Precuneus is vulnerable to early Aβ accumulation in preclinical AD

Several studies have earlier examined the expression of neuroinflammatory genes in the prefrontal or entorhinal cortex of the patients with AD using single nucleus transcriptome analysis [18–20]. However, there are only a few transcriptomic studies focusing on the precuneus [21, 22], the region of the brain, where Aβ accumulation is observed in preclinical AD patients [23, 24]. Therefore, the transcriptomic analysis of precuneus is of particular interest, as it can provide novel insights into the response of microglia to Aβ in early AD. Hence, we performed a gene expression analysis using RNA-seq in the precuneus of early AD patients, in parallel with AD model mice.

## Main text

### The reduced expression of microglial homeostatic genes was correlated with the degree of neuronal cell loss

To examine the correlation between the loss of homeostatic microglia functions and the degree of neuronal cell loss, we analyzed the expression levels of 68 homeostatic microglial genes in microglia isolated by magnetic-activated cell sorting from three representative mouse models exhibiting different severities of neurodegeneration.

- First model was an AD model, homozygous *App*^*NL-G-F/NL-G-F*^ knock-in mouse carrying *App* gene with humanized Aβ sequence having three familial AD mutations: Swedish (KM670/671NL), Beyreuther/Iberian (I716F), and Arctic (E693G), exhibiting amyloid pathology and neuroinflammation in an age-dependent manner without neuronal cell loss or NFTs [25].
- Second model was a tauopathy model, rTg4510 mouse overexpressing a mutant form of human Tau carrying the P301L mutation of familial frontotemporal dementia, resulting in brain atrophy with neuronal loss and neuroinflammation [26].
- The third model was an ALS model, SOD1^G93A^ mouse expressing a mutant form of human SOD1 carrying the G93A mutation of familial ALS, displaying motor neuron loss and neuroinflammation [27].

**Fig. 1:**
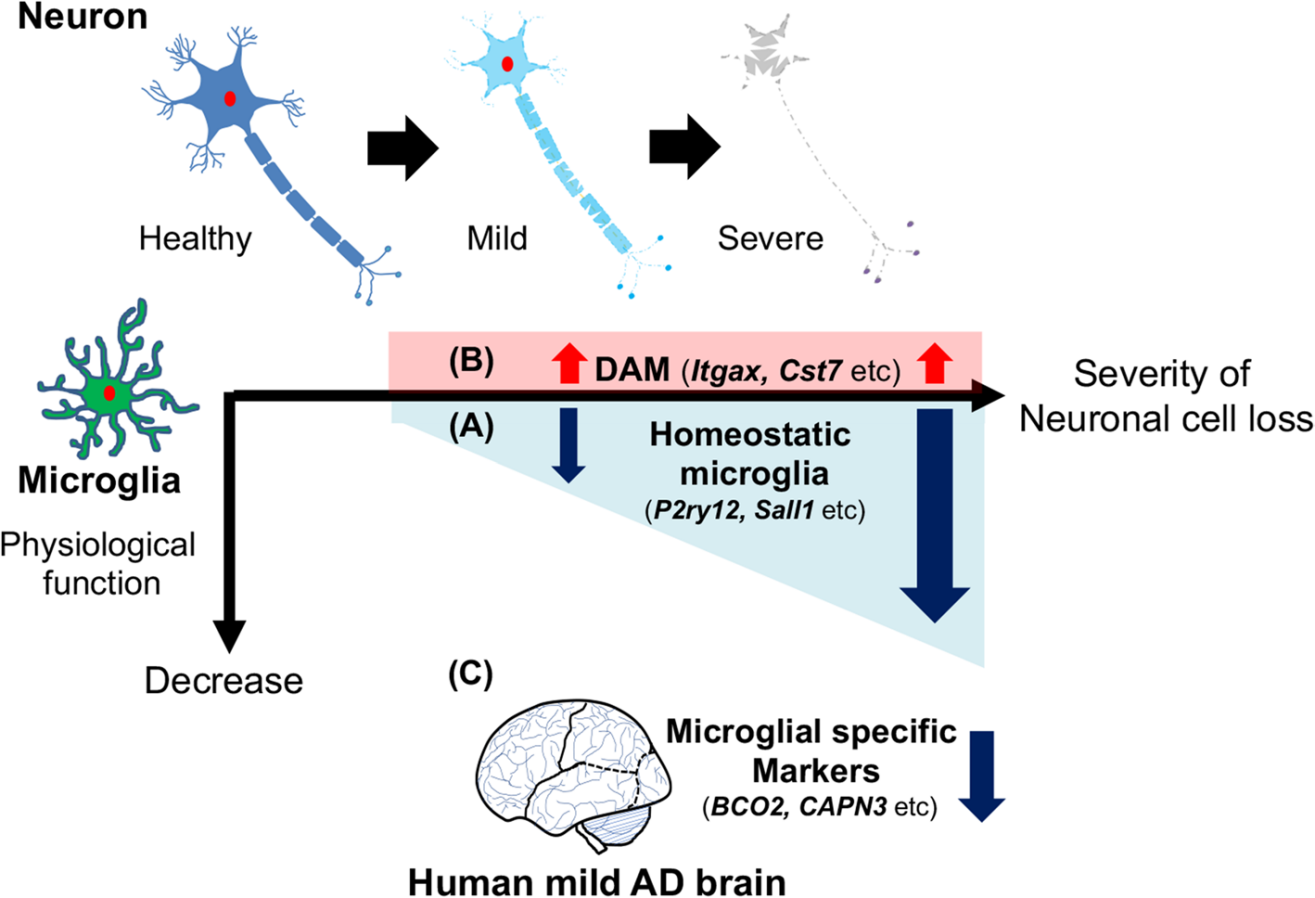
A schematic illustration for the microglial signature and their relevance to the severity of neurodegeneration in AD. **A** Microglial gene signature in mice revealed that a loss of homeostatic microglial function is associated with the degree of neuronal cell loss. **B** Most of the DAM genes were uniformly upregulated in each model. **C** In humans, a gene expression analysis of the precuneus of mild AD pathology also indicates a loss of microglial function induced by mild amyloid pathology

In this study, we used *App*^*NL-G-F/NL-G-F*^ mouse as a model of mild disease and rTg4510 and SOD1^G93A^ mice as severe models of neurodegenerative diseases. We found that microglia isolated from rTg4510 cortices and SOD1^G93A^ spinal cords distinctly showed reduced expressions of homeostatic microglial genes, whereas these reductions were not observed in microglia from *App*^*NL-G-F/NL-G-F*^ cortices. Moreover, the decrease in their expressions in SOD1^G93A^ spinal microglia was more obvious than those in rTg4510 cortical microglia. Since SOD1^G93A^ mice exhibit faster progression of neurodegeneration than rTg4510 mice, this reduced expression might be attributed to the severity of neuronal loss. A combination of these two observations suggests that the reduced expression levels of homeostatic microglial genes could be related to the degree of neuronal loss. In particular, we found that *P2ry12* and *Sall1* expressions were strongly associated with the degree of neuronal loss [28]. Previous reports indicate that P2RY12 is essential for regulating microglial activation via extracellular nucleotides derived from dead cells [29, 30], and that SALL1 encoding a transcriptional regulator inhibits a reactive microglia phenotype and promotes a physiological surveilling phenotype [31]. These reports indicate that these proteins influence the maintenance of the CNS homeostasis. Intriguingly, the reduction of homeostatic microglial gene expressions was also observed in both model mice of cuprizone-induced demyelination and the patients with multiple sclerosis, implying that the demyelination may act as a trigger for it perhaps prior to the neuronal loss [32]. Therefore, the loss of microglial homeostatic functions may be one of the primary hallmarks of progressive neurodegeneration, and their maintenance is presumably beneficial and might be a potential target for the treatment of neurodegenerative diseases (Fig. 1A) [28].

### No association between the expression levels of DAM genes and the severity of neurodegeneration

In order to examine the expressions of DAM genes and the correlation between their expression levels and severities of neuronal cell loss in these mouse models, we compared the expression profiles of the162 DAM genes in the microglia of each model (*fold change > 1.5, false discovery rate (FDR)* *< 0.05*) [12]. While almost all of the DAM genes, including *Itgax* and *Csf1*, were uniformly upregulated in the microglia of each model, the expression levels of twelve genes including *Apoe*, *Axl*, and *Cybb* seemed to have a correlation with the severity of neuronal cell loss [28]. These results indicate that increased expressions of DAM genes were not correlated with the degree of neuronal cell loss. Among the twelve identified genes, previous studies have indicated that *Apoe*, known as a major risk factor for AD, is essential for inducing DAM [14, 33]. We found that the expression level of *Apoe* negatively correlated with both homeostatic microglial genes and the neuronal survival. However, our results did not indicate any overall correlations between the expression levels of majority of the DAM genes, homeostatic microglial genes, and the degree of neuronal loss. Therefore, these findings suggest that the progression of neurodegeneration is not directly linked to DAM genes, but a few molecules including APOE may be involved in both homeostatic microglial dysfunctions as well as the progression of neurodegenerative diseases (Fig. 1B) [28].

### Transcriptomic analysis revealed moderate dysfunctions of microglia and oligodendrocytes in human precuneus with AD pathology

To uncover the changes in gene expression in the human brain with an early amyloid pathology, we performed RNA-seq analysis on the precuneus region derived from brains of deceased individuals. The subjects were either neuropathologically diagnosed with mild AD or controls. These samples were selected based on the Braak neuropathological staging [34] as follows:

- Fourteen non-AD brains (Braak stage: 0-A for senile plaque (SP) and 0–2 for NFT)
- Eleven early AD brains (Braak stage: C for SP and 3–4 for NFT)

All subjects with mild AD pathology had no apparent family history of dementia, implying that they were sporadic cases. We identified 643 deregulated genes, consisting of 127 upregulated and 516 downregulated genes in AD precunei (|fold change|> 1.5, *q* < 0.05). We compared the gene expression profiles of each precuneus with lists of neuron-, microglia-, astrocyte-, and oligodendrocyte-specific marker genes. Although the expressions of the representative microglial activation marker genes, such as *AIF1*, *CD68*, and *LGALS3*, were not altered, those of microglial-specific genes such as *BCO2*, *CAPN3*, small G-protein-associated genes (*RASGRP3* and *RAPGEF5)*, *PACC1*, also known as *TMEM206*, and *P2RX7*, were significantly decreased in AD precunei compared with controls. Moreover, the expressions of oligodendrocyte marker genes such as *MBP*, *MAG*, *CLDN11*, *MOG*, and *CNP* were also significantly decreased in AD precunei. However, there were no differences in the expressions of representative neuron and astrocyte marker genes. Our analyses revealed that the early amyloid pathology induces moderate dysfunctions of microglia and oligodendrocytes in the AD precuneus. Of note, the pathogenic role of deregulated oligodendrocytes is an open question. Our data suggest that both microglia and oligodendrocyte dysfunction may contribute to brain dysfunction induced by early AD pathology (Fig. 1C) [28].

### There were subtle alterations in DAM gene expressions in the AD precuneus

Although reactive microgliosis with neuroinflammation is one of the neuropathological characteristics of the AD brain [3, 35–37], it is not fully clear whether DAM gene expressions are deregulated in human precunei with early AD pathology. Our expression analysis revealed that only eight DAM genes (*APBB2*, *ARAP2*, *DHCR7*, *ENPP2*, *MYO1E*, *CD22*, *KCNJ2*, and *SLC44A1*) out of 162 DAM genes were deregulated in the AD precuneus, and unexpectedly, all of them were downregulated. In addition, the expression levels of representative DAM genes including *ITGAX*, *CST7*, *APOE*, *CSF1*, and *AXL* were marginally altered in these samples. Contrary to the prominent upregulation of DAM marker genes in the cortices of AD model mice, these expressions were hardly induced in human sporadic AD precunei [28]. Similar to our result, the previous transcriptomics studies have shown that few DAM genes were induced in human AD brains [19, 20, 38, 39]. The difference in reactivities to Aβ between human and mouse microglia was also reported [40]. These pieces of evidence suggest the discordance in microglial transcriptome signature between humans and mice in AD conditions. In addition, recent studies pointed out the low sensitivity of single nucleus RNA-seq to detect DAM genes in human postmortem brains [38, 41]. This low sensitivity may be attributed to the redistribution of DAM mRNAs to the cytosol or instability of DAM mRNAs. Alternatively, there is a possibility that microglial functions may be suppressed at the early stage of amyloid pathology in humans. Thus, to elucidate the significance of homeostatic microglia and DAM in human AD, further comparative transcriptomic analyses using the brain samples with early and advanced AD will be required.

### Identification of common altered genes in human AD precuneus and microglia of the mouse models of AD pathology

To understand the neuroinflammatory nature of early amyloid pathology in AD, we compared gene expression profiles among deregulated genes of AD precunei (|*fold change*| *> 1.2, q < 0.05*), those of *App*^*NL-G-F/NL-G-F*^ cortical microglia, and those of rTg4510 cortical microglia (|*fold change*| *> 1.5, q < 0.05, cut-off TPM > 5*). We identified eight upregulated genes and twenty-four downregulated genes in common among them. These upregulated genes include a chemokine (*CXCL10*) and interferon-induced genes (*STAT1*, *IFIT3*, *ISG15*), implying neuroinflammatory responses in AD precunei and AD mice. In addition, *App*^*NL-G-F/NL-G-F*^ mice shared forty-four deregulated genes (three upregulated and forty-one downregulated) with AD precunei. On the other hand, rTg4510 mice shared deregulated thirteen genes (two upregulated and eleven downregulated) with AD precuneus in common. Our data suggests that *App*^*NL-G-F/NL-G-F*^ and rTg4510 cortical microglia may represent distinct neuroinflammatory aspects relevant to AD pathologies [28]. Indeed, a previous study showed that microglia reactivities were different between amyloid and Tau pathologies [42]. In-depth analyses of molecular mechanisms in the identified molecules will lead to a better understanding of glial dysfunctions and identification of novel therapeutic targets.

## Conclusions

In the present review, we have discussed the microglial subtypes and molecular pathogenesis of microglia in AD. The results from our study indicate a correlation between glial phenotypes and the severity of neurodegeneration. Loss of microglial homeostatic functions might have a significant impact on the progression of neurodegeneration. Therefore, maintenance of homeostatic microglial functions is thought to be important in early AD. Although a validation of deregulated microglial genes at the protein level will be required, this review will provide important resources to better understand the role of glial dysfunction in AD. Further understanding of the molecular pathology and function of microglia during AD progression will contribute to development of AD therapies targeting neuroinflammation.

## Data Availability

Not applicable.

## Abbreviations

Aβ: *β*-Amyloid
ACHE: Acetylcholinesterase
AD: Alzheimer’s disease
ALDH1L1: Aldehyde dehydrogenase 1 family member L1
ALS: Amyotrophic lateral sclerosis
APBB2: Amyloid-beta precursor protein-binding family B member 2
APOE: Apolipoprotein E
App: Amyloid precursor protein
ARAP2: ArfGAP with RhoGAP domain, ankyrin repeat, and PH domain 2
ARM: Activated response microglia
AXL: AXL receptor tyrosine kinase
CSF1: Colony-stimulating factor 1
CST7: Cystatin F
Cxcl10: C-X-C motif chemokine ligand 10
Cybb: Cytochrome b-245 beta chain
DAM: Disease-associated microglia
DHCR7: 7-Dehydrocholesterol reductase
ENPP2: Ectonucleotide pyrophosphatase/phosphodiesterase 2
FDR: False discovery rate
GFAP: Glial fibrillary acidic protein
GRIN1: Glutamate ionotropic receptor NMDA type subunit 1
GRIN2B: Glutamate ionotropic receptor NMDA type subunit 2B
IFIT3: Interferon-induced protein with tetratricopeptide repeats 3
ISG15: ISG15 ubiquitin-like modifier
ITGAX: Integrin subunit alpha X
KCNJ2: Potassium inwardly rectifying channel subfamily J member 2
MAP2: Microtubule-associated protein 2
MGnD: Microglial neurodegenerative phenotype
MYO1E: Myosin IE
NFT: Neurofibrillary tangle
PET: Positron emission tomography
P2RY12: Purinergic receptor P2Y12
PTPRD: Protein tyrosine phosphatase receptor type D
S100B: S100 calcium-binding protein B
SALL1: Spalt-like transcription factor 1
SLC44A1: Solute carrier family 44 member 1
SOD: Superoxide dismutase
SP: Senile plaque
STAT1: Signal transducer and activator of transcription 1
SYP: Synaptophysin
TNFSF10: TNF superfamily member 10
Trem2: Triggering receptor expressed on myeloid cells 2
